# The Book World of Medicine and Science

**Published:** 1902-11-15

**Authors:** 


### The Book World of Medicine and Science.

Hydatid Disease of the Lungs. By A. A. Lendon, M.D.Lond., Lecturer on Obstetrics and Clinical Lecturer on Diseases of Children in the University of Adelaide, etc. (Baillière, Tindall and Cox. August, 1902. Demy 8vo. Three plates. 5s. net.)

In London, and in most places in England, hydatid disease is comparatively rare and, *a fortiori*, hydatid disease of the lungs is extremely rare. We have, therefore, every reason to be most grateful to Dr. Lendon for this little volume, in which he has embodied the results of his extensive experience of the disease in Australia. His method of dividing the disease into stages, such as the stage of the unruptured cyst, the stage of the rupture of the cyst, the stage of the ruptured cyst, etc., is, as he admits, not altogether satisfactory, because some so-called stages are really alternative events. Still we agree with him, that this method is convenient for the purposes of description. The question of diagnosis is gone into most carefully, and everyone will find that Dr. Lendon's experiences and opinions are of great use to him. It is evident, however, that in spite of all the book contains, the diagnosis of hydatids in the lungs must always be extremely difficult, until the characteristic fluid, membrane, or hooklets are detected. This must be especially the case in places where hydatid disease is rare; indeed, in some of the cases quoted the diagnosis of the physician appears to have been guided by the prevalence of the disease in the neighbourhood. As regards treatment it is needless to say that Dr. Lendon advocates free incision in preference to aspiration. He quotes cases to show that when the fluid is escaping through the aspirating needle and the cyst is collapsing, the cyst wall is likely to break up, and, as the contents cannot rapidly escape through the needle, they are almost certain to be coughed into the bronchial tubes, and the patient runs a great risk of being drowned by the fluid or choked by pieces of the cyst wall. On the other hand, if a free incision had been made, the cyst contents could easily escape through it.

Small-pox: How it is Spread and How it May be Prevented. By James Wallace, M.A., M.D.Aberdeen. (Henry J. Glaisher, 57 Wigmore Street, Cavendish Square, W. June, 1902. Sixty-nine pages. 2s. 6d. net.)

Dr. Wallace's account of the outbreak of small-pox at Warrington in 1892–1893 may be read with the greatest interest both by medical practitioners and by laymen, and the value of the little book is much enhanced by the comparison which he is able to draw between this particular epidemic and one of the same disease which occurred in the same town in 1773. It is almost unnecessary to say that the book materially contributes towards the evidence in favour of vaccination and revaccination. Perhaps the most remarkable bit of evidence is that, whereas in 1773 all the deaths (which amounted to 40 per cent. of the cases) occurred in children under nine years of age, in 1892 no one under the age of nine died, a conclusive proof that an infant, when properly vaccinated, is protected against small-pox for eight or nine years. Another interesting point is that the statistics clearly show that, if a person has been vaccinated as an infant, he is, if exposed to infection, most likely to contract small-pox between the ages of 20 and 30, showing that immunity does not last much more than 20 years, and further showing the necessity for revaccination during the second decade of life. It is a pity to over-argue a good case, and Dr. Wallace might have left out, with advantage, the statement that there is no improvement in our treatment of small-pox; for, although no specific remedy has been discovered, the modern methods of supplying fever patients with abundance of fresh air, with food which is easily assimilated, and with stimulants, contrasts favourably with the older methods of venesection and purgation and would tend to lessen the mortality. It is important to note that the evidence supplied by the epidemic in 1892 is against the idea that small-pox is disseminated through the air from a centre at which a number of cases are collected.

International Clinics. A Quarterly Volume of Illustrated Clinical Lectures and Especially Prepared Articles. Edited by Henry W. Cattell, A.M., M.D.Phila., U.S.A. Vol. I. Twelfth Series. (London: J. B. Lippincott Company. 1902. Price 10s. 6d. net.)

"International Clinics" is unique. It is a strong bond between English-speaking practitioners of medicine. Although edited from Philadelphia it is supported by leading members of the medical profession throughout the world. Such a periodical is not only a credit to American enterprise but does much to maintain a friendly relationship with those across the Atlantic. The present number is full of interest and rich in variety; but of the twenty contributors we note that only two are from this country. Among some of the special features notice should be made of the attractive biographical sketches of such eminent living medical men as Weir-Mitchell and J. A. Wyeth. In this old-fashioned country it is difficult to break away from ancient customs and venerable traditions, but young America apparently sees nothing unseemly in presenting a photograph of Dr. S. Weir-Mitchell at his clinic for nervous diseases, or the portrayal of Dr. Wyeth conducting an operation at the New York Polyclinic Hospital. Many of the articles are of much practical value. Dr. Arthur V. Meigs writes on the use of opium in daily practice; Professor Boas, of Berlin, treats of habitual constipation; Professor H. Hallopean deals with the treatment of acne; Dr. Simon has an important study on the significance of basophilic granules in red corpuscles, with special reference to their occurrence in chronic lead poisoning; Professor Hemmeter discusses gastro-intestinal auto-intoxication; and Dr. A. Boissard, of Paris, deals with the relative advantages of symphyseotomy and Cæsarian section. There is also an excellent summary of the progress of medicine during 1901. It is impossible in the scope of a short notice to indicate the wide range of subjects dealt with, but it may be safely said that no one, whatever his speciality, can fail to be interested in the contents of this volume. It should prove of particular service to the busy practitioner desirous of keeping himself well abreast of the times.

